# Diaphragm assessment in mice overexpressing phospholamban in slow‐twitch type I muscle fibers

**DOI:** 10.1002/brb3.470

**Published:** 2016-04-22

**Authors:** Val Andrew Fajardo, Ian Curtis Smith, Eric Bombardier, Paige J. Chambers, Joe Quadrilatero, Allan Russell Tupling

**Affiliations:** ^1^ Department of KinesiologyUniversity of WaterlooWaterlooONCanada

**Keywords:** Centronuclear myopathy, fiber types, phospholamban, sarcolipin, SERCA

## Abstract

**Aims:**

Phospholamban (PLN) and sarcolipin (SLN) are small inhibitory proteins that regulate the sarco(endo)plasmic reticulum Ca^2+^‐ATPase (SERCA) pump. Previous work from our laboratory revealed that in the soleus and gluteus minimus muscles of mice overexpressing PLN (*Pln* ^OE^), SERCA function was impaired, dynamin 2 (3–5 fold) and SLN (7–9 fold) were upregulated, and features of human centronuclear myopathy (CNM) were observed. Here, we performed structural and functional experiments to evaluate whether the diaphragm muscles of the *Pln* ^OE^ mouse would exhibit CNM pathology and muscle weakness.

**Methods:**

Diaphragm muscles from *Pln* ^OE^ and WT mice were subjected to histological/histochemical/immunofluorescent staining, Ca^2+^‐ATPase and Ca^2+^ uptake assays, Western blotting, and *in vitro* electrical stimulation.

**Results:**

Our results demonstrate that PLN overexpression reduced SERCA's apparent affinity for Ca^2+^ but did not reduce maximal SERCA activity or rates of Ca^2+^ uptake. SLN was upregulated 2.5‐fold, whereas no changes in dynamin 2 expression were found. With respect to CNM, we did not observe type I fiber predominance, central nuclei, or central aggregation of oxidative activity in diaphragm, although type I fiber hypotrophy was present. Furthermore, *in vitro* contractility assessment of *Pln* ^OE^ diaphragm strips revealed no reductions in force‐generating capacity, maximal rates of relaxation or force development, but did indicate that ½ relaxation time was prolonged.

**Conclusions:**

Therefore, the effects of PLN overexpression on skeletal muscle phenotype differ between diaphragm and the postural soleus and gluteus minimus muscles. Our findings here point to differences in SLN expression and type I fiber distribution as potential contributing factors.

## Introduction

Centronuclear myopathies (CNM) are a group of congenital myopathies that, along with general muscle weakness, display increased central nuclei, type I fiber predominance and hypotrophy, and central aggregation of oxidative activity upon muscle biopsy (Sharma et al. 2009; Romero 2010). Phospholamban (PLN) is a small (52 amino acid) protein that physically interacts with and inhibits the sarco(endo)plasmic reticulum Ca^2+^‐ATPase (SERCA) pump (Morita et al. 2008). Recently, we found that the soleus and gluteus minimus muscles from mice overexpressing PLN (*Pln* ^OE^) in their slow‐twitch type I skeletal muscle fibers had impaired SERCA function and displayed the histopathological features associated with human CNM (Fajardo et al. 2015). Based on the appearance of radiating sarcoplasmic strands, type II fiber hypertrophy, and endomysial fibrosis, the CNM observed in the *Pln* ^OE^ mice resembles human autosomal dominant CNM and, to an extent, *RYR*‐related and *TTN*‐related CNM (Jungbluth and Gautel 2014).

A recently published review highlighted that although diaphragm and ventilatory function have been examined in several murine models of myopathy including Pompe disease and Duchenne muscular dystrophy, among other neuromuscular diseases, similar reports are not available for animal models of CNM (Smith et al. 2014). Compared with the other two major CNM variants, X‐linked myotubular myopathy and autosomal‐recessive CNM, respiratory function in patients with autosomal dominant CNM, for the most part, is intact and patients live a normal lifespan (Bitoun et al. 2005; Jungbluth et al. 2008; Romero 2010; Smith et al. 2014). Thus, it was of interest to determine whether or not the diaphragm muscles of the *Pln* ^OE^ mouse, which more closely resembles autosomal dominant CNM, would exhibit CNM pathology and muscle weakness. Since in this model, PLN is overexpressed specifically in the type I fibers and the murine diaphragm normally consists of around 10% type I fibers (Green et al. 1984; Talmadge et al. 2004); we initially hypothesized that the diaphragm from *Pln* ^OE^ mice would display impaired SERCA function, muscle weakness, and the appearance of the histopathological features associated with human CNM.

## Materials and Methods

### Animals and tissue collection

The *Pln* ^OE^ mice were resuscitated from cryopreserved embryos by the mmRRC (00067‐MU, FVB/N background) to generate a breeding colony with WT FVB/N mice at the University of Waterloo. The *Pln* transgene was attached to the *β*‐MHC promoter so that these mice exhibit type I fiber‐specific PLN overexpression. A total of 15 male wild‐type (WT; 30.9 ± 1.2 g) and 16 male *Pln* ^OE^ (30.7 ± 0.9 g) 4–6‐month‐old mice were used in the study. Animals were housed in an environmentally controlled room with a standard 12:12‐h light–dark cycle and allowed access to food and water ad libitum. Mice were sacrificed by cervical dislocation and diaphragm, soleus, and gluteus minimus muscles were extracted. We restricted analyses to the anterior‐lateral costal regions of the diaphragm muscles. Diaphragm strips were either used for the assessment of SERCA function, contractility, or histological/histochemical/immunofluorescence staining for the examination of central nuclei, central aggregation of oxidative activity and fiber type distribution and CSA. Nuclear and cytosolic fractions from the diaphragm, soleus, and gluteus minmus muscles were extracted as previously described (McMillan and Quadrilatero 2011). All animal procedures were reviewed and approved by the Animal Care Committee of the University of Waterloo and are consistent with the guidelines established by the Canadian Council on Animal Care.

### SERCA activity and Ca^2+^ uptake

Ca^2+^‐dependent SERCA activity was assessed in homogenates prepared from mouse (WT and *Pln* ^OE^) diaphragm muscles over Ca^2+^ concentrations ranging from *p*Ca 7.0 to 4.8 at 37°C using a spectrophotometric plate reader assay that has been described previously (Duhamel et al. 2007). Briefly, 10 *μ*L of crude diaphragm homogenate was added to 5 mL cocktail buffer containing the reaction buffer (200 mmol/L KCl, 20 mmol/L HEPES, 15 mmol/L MgCl_2_, 10 mmol/L NaN_3_, 10 mmol/L phosphoenolpyruvate, 5 mmol/L ATP, 1 mmol/L EGTA, pH 7.0), 18 U/mL lactate dehydrogenase, 18 U/mL pyruvate kinase, 0.3 mmol/L NADH, in the presence of ionophore A23187 (Sigma C7522, 4 *μ*mol/L, St. Louis, MO). Total ATPase activity across the range of *p*Ca was determined as the rate of NADH disappearance over 30 min measured at 340 nm using a spectrophotometric plate reader (SPECTRAMAX plus; Molecular Devices, Toronto, ON, Canada). SERCA activity was then calculated by subtracting ATPase activity in the presence of a SERCA‐specific inhibitor, cyclopiazonic acid (40 *μ*mol/L) (Seidler et al. 1989), and SERCA activity‐*p*Ca curves were generated with GraphPad Prism^™^ (version 6, GraphPah Software, Inc. La Jolla, CA) by nonlinear regression curve fitting using an equation for a general cooperative model for substrate activation. Ca^2+^ uptake was measured in muscle homogenates at 37°C in the presence of the precipitating anion, oxalate, using the fluorescent dye Indo‐1 (50042; Biotium, Hayward, CA) and a spectrofluorometer (Ratiomaster™ System; Photon Technology International, Birmingham, NJ) equipped with a monochromator to control the excitation wavelength (355 nm) and two photomultiplier tubes to detect emitted light (405 and 485 nm) (Tupling and Green 2002). Rates of Ca^2+^ uptake were assessed at a free Ca^2+^ concentration of 1.0 *μ*mol/L.

### Antibodies

Primary antibodies against SERCA2a (2A7‐A1), PLN (2D12), NFATc1 (7A6), and dynamin 2 (PA5‐19800) were obtained from Pierce Antibodies. The primary antibody for calcineurin was obtained from Millipore (07‐1491). The primary antibody for SERCA1a (A52) was a kind gift from Dr. David MacLennan (University of Toronto) (Zubrzycka‐Gaarn et al. 1984). The primary antibody directed against sarcolipin (SLN) was generated by Lampire Biological Laboratories (Fajardo et al. 2013). The primary antibody against *α*‐actin (A4700) was obtained from Sigma Aldrich (St. Louis, MO). The primary antibodies against MHCI (BA‐F8), MHCIIa (SC‐71), and MHCIIb (BF‐F3) were obtained from Developmental Studies Hybridoma Bank (Schiaffino et al. 1989). These antibodies are highly specific for use in murine skeletal muscle, although the antibody for MHCIIa does cross‐react with MHCIIx in human tissue (Bloemberg and Quadrilatero 2012). Secondary antibodies for Western blotting, goat anti‐mouse IgG (peroxidase conjugated; sc‐2005) and goat anti‐rabbit IgG (peroxidase conjugated; sc‐2030) were obtained from Santa Cruz Biotechnology (Dallas, TX). Secondary antibodies for immunofluorescence staining, Alexa Fluor 350 anti‐mouse IgG_2b_ (A‐21140), Alexa Fluor 488 anti‐mouse IgG_1_ (A‐21121), and Alexa Fluor 555 anti‐mouse IgM (A‐21426), were obtained from Molecular Probes Thermo Fisher.

### Western blot analysis

Western blot analysis was performed to determine expression levels of SERCAs, SLN, PLN, and dynamin 2 in the diaphragm muscles from WT and *Pln* ^OE^ mice as previously described (Fajardo et al. 2015). In addition, calcineurin expression and NFAT nuclear localization in soleus, gluteus minimus, and diaphragm muscles were assessed. Samples were not boiled prior to electrophoresis, which allows for detection of monomeric and pentameric PLN content (Tupling et al. 2011). Electrophoretically separated proteins were transferred onto 0.2 *μ*m polyvinylidene difluoride (PVDF) membranes (PLN, 1:2000; SERCA1a, 1:10,000; SERCA2a, 1:2000; dynamin 2, 1:2000; NFATc1, 1:2000; calcineurin, 1:1000) or nitrocellulose membranes (SLN, 1:100) and then immunoprobed with their corresponding primary antibodies. Subsequently, membranes were washed and immunoprobed with either goat anti‐mouse IgG (horseradish peroxidase conjugated) with a 1:20,000 dilution for SERCA1a and SERCA2a, and a 1:2000 dilution for PLN, dynamin 2, and NFATc1; or a goat anti‐rabbit IgG (horseradish peroxidase conjugated) in a 1:2000 dilution for SLN and calcineurin. Antigen‐antibody complexes were detected by SuperSignal West Femto™ substrate (Pierce; Thermo Fisher Scientific Inc., Grand Island, NY) for SLN; Luminata Forte^™^ (Millipore, Billerica, MA) for PLN and SERCA2a; and ECL Western Blot Substrate (BioVision, MA) for SERCA1a, NFAT, calcineurin, and dynamin 2. Quantitation of optical densities was performed using GeneTools (Syngene, MD) and was normalized to total protein or *α*‐actin.

### Histological, histochemical, and immunofluorescence staining

Diaphragm muscles from WT and *Pln* ^OE^ mice were removed and embedded in O.C.T. compound (Tissue‐Tek, Sakura Finetek USA Inc., Torrance, CA), frozen in liquid nitrogen‐cooled isopentane, stored at −80°C, and cut into 10‐*μ*m‐thick cryosections with a cryostat (Thermo Fisher Scientific Inc., Grand Island, NY, United States) maintained at −20°C. To examine the percent of fibers containing centrally located nuclei and central aggregation of oxidative activity, hematoxylin and eosin (H&E) staining, and succinate dehydrogenase (SDH) activity were performed, respectively. Images were acquired with a brightfield Nikon microscope linked to a PixeLink digital camera and central nuclei counts were quantified with ImageJ software (National Institutes of Health, Bethesda, MD) using the cell counter plugin. One section of the diaphragm muscle from each mouse (*n* = 4 per genotype) was randomly selected and 793–1292 fibers were analyzed for central nuclei per section. For fiber type analysis (CSA and % distribution), immunofluorescence analysis of MHC expression with primary antibodies against MHCI, MHCIIa, and MHCIIb was performed. Details regarding the dilutions of the primary and secondary antibodies for fiber type analysis have been previously described (Bloemberg and Quadrilatero 2012). Slides were visualized with an Axio Observer Z1 fluorescent microscope equipped with standard red/green/blue filters, an AxioCam HRm camera, and AxioVision software (Carl Ziess, North York, ON, Canada). Details of the immunofluorescence procedures and analysis of MHC expression were previously described (McMillan and Quadrilatero 2011; Bloemberg and Quadrilatero 2012; Fajardo et al. 2013). Briefly, for fiber type distribution, one section of the diaphragm muscle from each mouse was randomly selected and all fiber types within that section were counted and sorted by fiber type (1126–3105 total fibers per section) using ImageJ's cell counter plugin. For analysis of fiber type area, 20 fibers of each type were randomly selected within a diaphragm section and the area of each fiber was determined and then averaged using ImageJ's area measurement tool after calibrating with the corresponding scale bar. This was repeated for five different WT and *Pln* ^OE^ mice, and the values reported represent the calculated averages from the five animals within the specific genotype.

### *In vitro* diaphragm contractile assessment

Diaphragm strips were isolated and immediately placed into a bath of oxygenated Tyrode's solution (95% O_2_, 5% CO_2_) containing 121 mmol/L NaCl_2_, 5 mmol/L KCl, 24 mmol/L NaHCO_3_, 1.8 mmol/L CaCl_2_, 0.4 mmol/L NaH_2_PO_4_, 5.5 mmol/L glucose, 0.1 mmol/L EDTA, and 0.5 mmol/L MgCl_2_, *p*H 7.3 (Bombardier et al. 2013) and were maintained at 25°C. Muscle strips were situated between flanking platinum electrodes driven by a biphasic simulator (Model 710B; Aurora Scientific, Inc., Aurora, ON, Canada) and electrically evoked muscle force was assessed across a range of stimulation frequencies from 1 to 100 Hz at optimum length for force production. Data were analyzed using Dynamic Muscle Control Data Acquisition software (Aurora Scientific, Inc). Specifically, peak isometric force amplitude (mN) was determined across the range of stimulation frequencies and measures of twitch kinetics: maximal rates of force development (+*d*F/*d*t) and relaxation (−*d*F/*d*t), ½ relaxation time (1/2 RT), and time to peak tension (TPT) were assessed. Peak isometric force was then normalized to the calculated CSA of the muscle strip (*m*/l*d) where *m* is the muscle mass, *l* is the length*,* and *d* is mammalian skeletal muscle density (1.06 mg/mm^3^) (Mendez 1960). A fatigue protocol (70 Hz for 350 ms every 2 sec for 5 min) was performed to determine the number of contractions required to reduce force to 60% of the force of the initial 70 Hz contraction.

### Statistics

All values are presented as means ± standard error. Statistical significance was set to *P < *0.05. Most comparisons between WT and *Pln* ^OE^ mice were performed using unpaired Student's *t*‐tests; however, a two‐way repeated measures ANOVA was used for force–frequency analysis of peak isometric force. A one‐way ANOVA with a Dunnet's multiple comparison post hoc test was used to compare PLN expression levels in WT gluteus minimus and soleus muscles with diaphragm.

## Results

### SERCA function and expression of Ca^2+^ handling proteins

**Figure 1:**
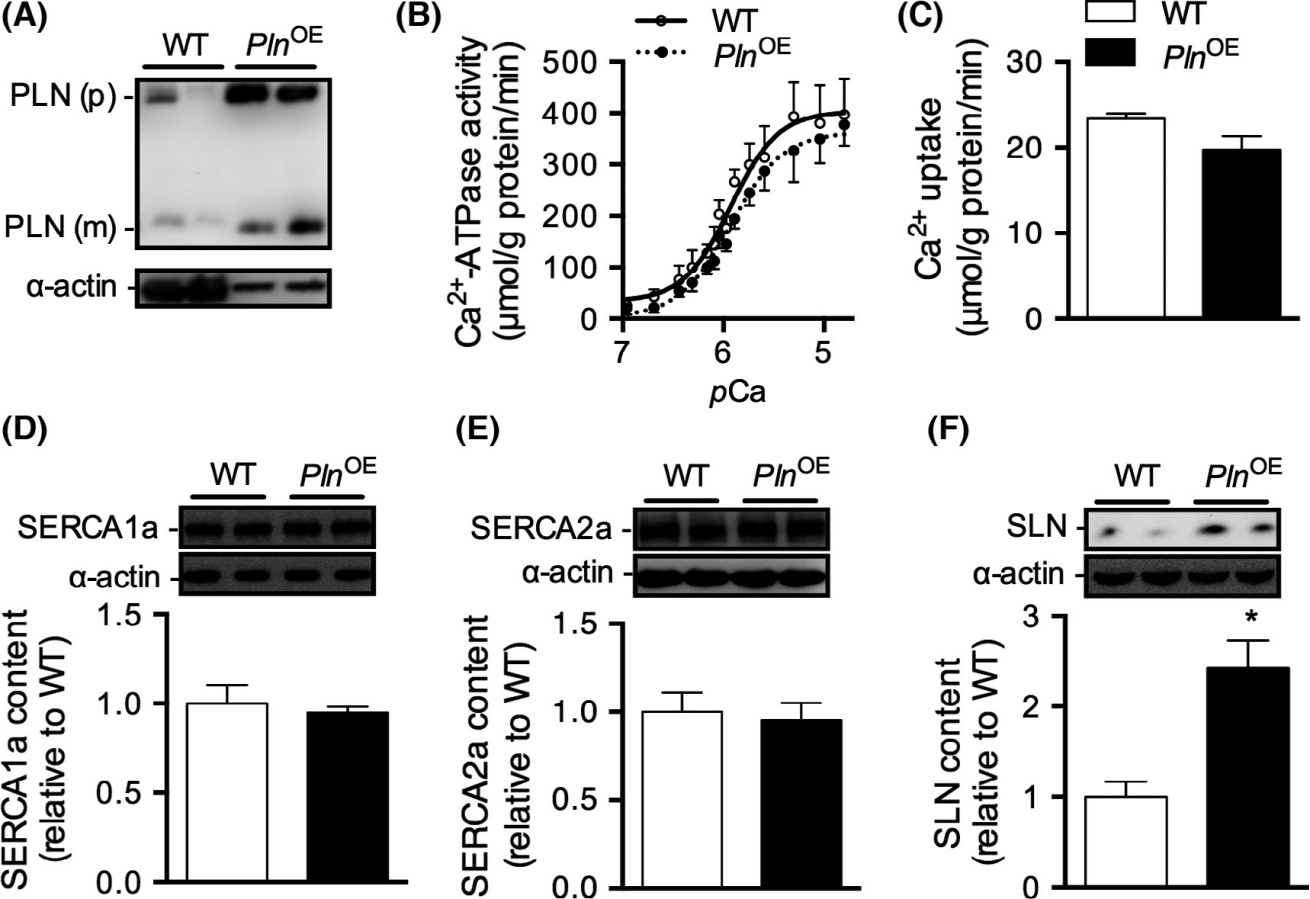
SERCA function in diaphragm muscles in *Pln* ^OE^ mice at 4–6 months of age. (A) Western blotting for PLN in WT and *Pln* ^OE^ mice from diaphragm muscle homogenates. For WT mice, 25 *μ*g of total protein was loaded, whereas only 2.5 *μ*g was required for *Pln* ^OE^ mice to detect PLN protein. (B) Ca^2+^‐ATPase activity‐*p*Ca curves in WT (*n* = 6) and *Pln* ^OE^ mice (*n* = 6) in the presence of the Ca^2+^ ionophore. (C) Ca^2+^ uptake assessed in diaphragm muscles from WT (*n* = 4) and *Pln* ^OE^ mice (*n* = 5). Western blotting for SERCA1a (D), SERCA2a (E), and SLN (F) in soleus from WT and *Pln* ^OE^ mice (*n* = 4–5 per genotype). Total protein loaded was 1 *μ*g, 4 *μ*g, and 25 *μ*g for SERCA1a, SERCA2a, and SLN, respectively. Actin was used as a loading control and all values are expressed relative to WT. For, A, D, E the two lanes for WT and *Pln* ^OE^ correspond to samples from two different WT and two different *Pln* ^OE^ mice, respectively. *P ≤ 0.05 versus WT using Student's *t*‐test. All values are presented as mean ± standard error.

Phospholamban overexpression was evident in the diaphragm homogenates from *Pln* ^OE^ mice as only 2.5 *μ*g of total protein was required to detect PLN compared to the 25 *μ*g required in the WT diaphragm (Fig. 1A). Specifically, measurements of optical density relative to actin indicate a 13‐fold and 22‐fold overexpression of PLN monomer and pentamer, respectively, compared with WT. PLN overexpression in diaphragm muscles was associated with a rightward shift in the activity‐*p*Ca curves (Fig. 1B) and a significant increase in *K*_*Ca*_ compared to WT (Table 1, *P* = 0.02). However, maximal SERCA activity was not significantly different between genotypes (Table 1, *P* = 0.45). We did observe lower rates of Ca^2+^ uptake in the *Pln* ^OE^ diaphragm homogenates compared to WT (Fig. 1C), however, this only approached statistical significance (*P *=* *0.08). Western blot analysis revealed no differences in the expression of SERCA1a (Fig. 1D) or SERCA2a (Fig. 1E) between WT and *Pln* ^OE^ mice. SLN expression was found to be upregulated nearly 2.5‐fold in the diaphragm homogenates from *Pln* ^OE^ mice compared to WT (Fig. 1F, *P* < 0.001).

**Table 1:** SERCA activity in mouse diaphragm muscles from WT and *Pln* ^OE^ mice at 4–6 months of age

Genotype	*V*_max_ (*μ*mol/g of protein/min)	*K*_Ca_	Δ*K*_Ca_
WT	409.1 ± 29.8	5.98 ± 0.02	–
*Pln* ^OE^	382.1 ± 17.3	5.90 ± 0.02^a^	0.08

Values are means ± standard error. Homogenates were isolated from WT (*n* = 6) and *Pln* ^OE^ (*n* = 6) mouse diaphragm muscles and were analyzed for Ca^2+^‐ATPase activity over Ca^2+^ concentrations ranging from pCa 7 to pCa 4.8 to obtain *K*_Ca_. *K*_*Ca*_ is the Ca^2+^ concentration required to attain the half‐maximal Ca^2+^‐ATPase activity rate and is expressed in *p*Ca units. Δ*K*_Ca_ is the difference in *K*_Ca_ between genotypes.

a Significantly different from WT using Student's *t*‐test, *P < *0.05.

### Assessment of CNM features in diaphragm muscles from *Pln* ^OE^ mice

**Figure 2:**
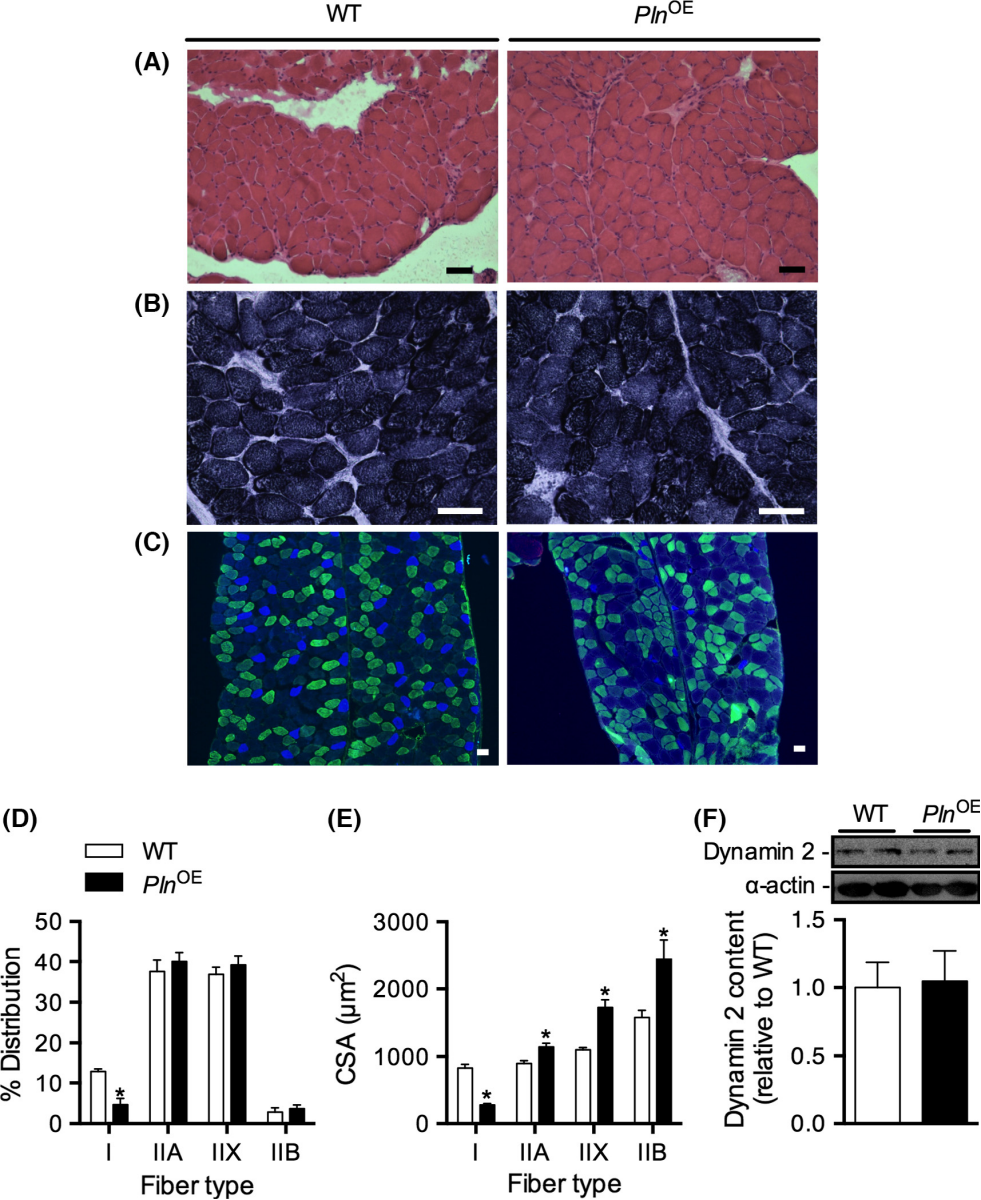
Diaphragm muscles from *Pln* ^OE^ mice do not display CNM. Representative diaphragm cryosections from *Pln* ^OE^ and WT mice after H&E staining (A), SDH staining (B), immunofluorescence staining for fiber type analysis (C) (*n* = 5 per genotype for all staining protocols). Cross sections were stained with MHC antibodies to identify type I (blue), type IIA (green), type IIB (red), and type IIX (unstained). (D) Quantitative analysis of fiber type distribution in diaphragm muscles from WT and *Pln* ^OE^ mice at 4–6 months of age (*n* = 5 per genotype). (E) Quantitative analysis of fiber type cross‐sectional area (CSA) in diaphragm muscles from WT and *Pln* ^OE^ mice at 4–6 months of age (*n* = 5 per genotype). (F) Dynamin 2 expression in diaphragm homogenates from WT and *Pln* ^OE^ mice (*n* = 6 per genotype). Total protein loaded for dynamin 2 was 7.5 *μ*g. *P ≤ 0.05 versus WT using Student's *t*‐test. All values are presented as mean ± standard error. Scale bars in (A–C) are set to 50 *μ*m.

H&E and SDH staining of diaphragm cryosections revealed no elevations in the percentage of fibers showing central nuclei (WT, 1.2 ± 0.2% vs. *Pln* ^OE^, 1.2 ± 0.3%, *P *=* *0.86) or evidence of central aggregation of oxidative activity in the *Pln* ^OE^ diaphragm (Fig. 2A and B). A significant reduction in the percentage of type I fibers was found (Fig. 2C and D, *P* < 0.001) and although individually the type II fibers (IIA, IIX, IIB) were not different between genotypes, there was a significant increase in the collective percentage of all type II fibers in the *Pln* ^OE^ diaphragm (WT, 86.5 ± 0.7% vs. *Pln* ^OE^, 95.0 ± 1.8%, *P *<* *0.001). Furthermore, we observed a significant reduction (*P *<* *0.001) in type I fiber CSA and a significant increase in type IIA (*P *=* *0.01), type IIX (*P *<* *0.001), and type IIB (*P *<* *0.001) CSA in the *Pln* ^OE^ diaphragm (Fig. 2E). Western blotting for dynamin 2 expression in diaphragm revealed no significant differences between WT and *Pln* ^OE^ mice (Fig. 2F, *P* = 0.87).

### Diaphragm contractility

**Figure 3:**
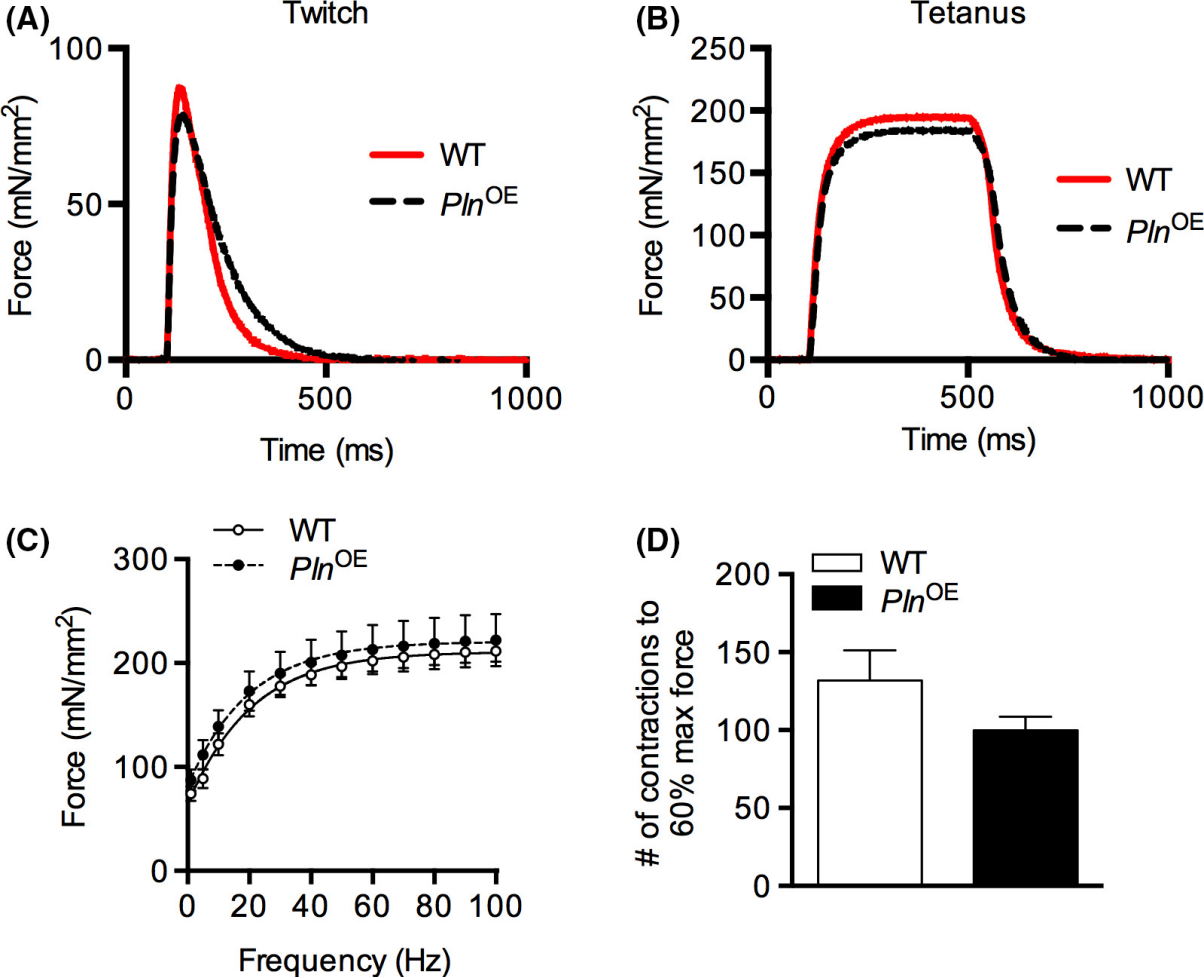
Diaphragm contractility in *Pln* ^OE^ and WT mice at 4–6 months of age. (A) Representative twitch (A) and tetanic (B) force tracings normalized to CSA from WT and *Pln* ^OE^ diaphragm strips. (C) Force–frequency curve analysis in diaphragm strips from WT and *Pln* ^OE^ mice. (D) Number of contractions to reduce force to 60% of initial isometric force when isolated diaphragm strips underwent a fatiguing stimulation protocol (70 Hz for 350 ms every 2 sec for 5 min). For C and D, all values are presented as mean ± standard error, *n* = 6 per genotype.

Representative twitch and tetanic (100 Hz) force tracings illustrate no significant differences in force production between WT and *Pln* ^OE^ diaphragm strips (Fig. 3A and B). In addition, there were no differences in maximal rates of relaxation (−dF/d*t*: WT, 0.73 ± 0.04 vs. *Pln* ^OE^, 0.68 ± 0.07, mN/ms/mm^2^, *P *=* *0.49) or maximal rates of force development (+*d*F/*dt*: WT, 5.04 ± 0.51 vs. *Pln* ^OE^, 5.87 ± 0.68 mN/ms/mm^2^, *P *=* *0.34). Correspondingly, there were no differences in TPT between genotypes (WT, 39. 2 ± 1.9 vs. *Pln* ^OE^, 38.5 ± 1.7, ms, *P *=* *0.78, *n* = 6 per genotype); however, there was a significant increase in ½ relaxation time in the *Pln* ^OE^ diaphragm compared with WT (WT, 78.0 ± 5.3 vs. *Pln* ^OE^ 95.8 ± 3.8, ms, *P *=* *0.02, *n* = 6 per genotype). Interestingly, across submaximal and maximal frequencies, *Pln* ^OE^ diaphragm strips generated more specific force compared to WT but the difference was not significant (Fig. 3C). Finally, in response to a fatiguing stimulation protocol, *Pln* ^OE^ diaphragms required on average 100 ± 8 contractions to reach 60% maximum isometric force at 70 Hz compared to 132 ± 19 in the WT diaphragm; however, this difference was not statistically significant (Fig. 3D, *P* = 0.16).

## Discussion

Studies examining diaphragm function in animal models of CNM are limited as recently highlighted in a review by Smith Goddard, and Childers (Smith et al. 2014). However, in a study published in the same year, Cowling et al. (2014) showed histopathological abnormalities with more atrophic fibers containing mislocalized nuclei in the diaphragm muscles from *mtm1* ^−/y^ mice, a mouse model which accurately recapitulates human X‐linked myotubular myopathy (Buj‐Bello et al. 2002). Correspondingly, *mtm1* ^−/y^ mice have a very short lifespan (6–14 weeks) (Buj‐Bello et al. 2002) which is consistent with the severe respiratory complications often leading to early death reported in human cases of myotubular myopathy (Jungbluth et al. 2008). We have recently reported that the *Pln* ^OE^ mouse histopathologically resembles human autosomal dominant CNM (Fajardo et al. 2015), which, in contrast to myotubular myopathy, is generally less severe, and patients often have a normal lifespan with very few respiratory complications (Bitoun et al. 2005; Jungbluth et al. 2008; Romero 2010). Here, we have shown that the *Pln* ^OE^ diaphragm exhibits type I fiber hypotrophy but does not display type I fiber predominance, increased central nuclei, central aggregation of oxidative activity, or muscle weakness. In agreement with the differences in disease severity found in the diaphragm between mouse models, the *Pln* ^OE^ mice live much longer than *mtm1* ^−/y^ mice, albeit, shorter than their WT littermates (Fig. S1).

Previous studies have shown that elevated dynamin 2 in skeletal muscle can lead to features of CNM (Cowling et al. 2011; Liu et al. 2011) while reducing its expression can improve muscle function and alleviate murine CNM pathology (Cowling et al. 2014). Since, dynamin 2 is upregulated 3–5 fold in the *Pln* ^OE^ soleus and gluteus minimus (Fajardo et al. 2015) but not diaphragm (Fig. 2F), it is possible that differences in dynamin 2 expression could explain the lack of CNM phenotype in the *Pln* ^OE^ diaphragm. How PLN overexpression and impaired SERCA function leads to increased dynamin 2 in the affected muscles is unclear. Furthermore, although dynamin 2 has been implicated in the pathology of murine CNM, its role in human CNM is less established since we have recently observed a 35% reduction in its expression in muscle biopsies from CNM patients (Fajardo et al. 2015) while others have seen a twofold increase in patients with myotubular myopathy (Cowling et al. 2014).

Our findings of minimal histopathological defects within the *Pln* ^OE^ diaphragm despite PLN overexpression may argue against a potential role of elevated PLN in CNM pathology. An important difference between diaphragm and the postural limb muscles in the *Pln* ^OE^ model is that SERCA function was not impaired in the diaphragm to the extent we observed in the soleus and gluteus minimus muscles (Fajardo et al. 2015). Maximal SERCA activity was not reduced in the *Pln* ^OE^ diaphragm compared to WT and rates of Ca^2+^ uptake were only trending to be lower in the *Pln* ^OE^ diaphragm compared to WT (−15%), whereas Ca^2+^ uptake measured at the same *p*Ca was significantly reduced in the *Pln* ^OE^ soleus (−75%) and gluteus minimus muscles (−25%) (Fig. S3). This may be surprising given the 13‐fold overexpression of monomeric PLN in the *Pln* ^OE^ diaphragm compared with the previously observed 6.3‐fold and 6.7‐fold overexpression found in the soleus and gluteus miminus, respectively (Fajardo et al. 2015). However, since these measures are expressed relative to WT, it appears that the differences in PLN overexpression across muscle types can be accounted for by a fivefold lower monomeric PLN expression in the WT diaphragm compared with soleus and gluteus minimus muscles (Fig. S2). In our view, the simplest explanation for the muscle differences in this model is that WT diaphragm has the lowest proportion of type I fibers (~12%) compared with soleus and gluteus minimus, which is even lower in *Pln* ^OE^ (~6%); therefore, the inhibitory effects of PLN overexpression on SERCA function would be constrained to the few type I fibers present in diaphragm.

Despite near normal maximal activity, SERCA's apparent affinity for Ca^2+^ was reduced in the *Pln* ^OE^ diaphragm as indicated by a rightward shift in the activity‐*p*Ca curve and a significantly higher *K*_*Ca*_. Reduced Ca^2+^ sensitivity of SERCA activity would be expected with PLN overexpression in type I fibers but could also be due to increased SLN expression. SLN is a well‐known structural and functional homolog of PLN (Odermatt et al. 1998; Tupling et al. 2011; Fajardo et al. 2013; Gorski et al. 2013) and, together, they have been shown to form a superinhibitory ternary complex with SERCA that not only reduces SERCA's affinity for Ca^2+^, but also the maximal rates of SERCA activity (Gorski et al. 2013) and Ca^2+^ uptake (Asahi et al. 2002). Interestingly, compared to the soleus and gluteus minimus, where SLN protein was upregulated ninefold and sevenfold, respectively (Fajardo et al. 2015), SLN in the diaphragm was only elevated 2.5‐fold, providing another possible mechanism which may contribute to the differences in severity of SERCA dysfunction across skeletal muscles in the *Pln* ^OE^ mouse. Finally, differences in SERCA's apparent affinity for Ca^2+^ may be related to differences in SERCA isoform expression. In a previous study using human single fibers, it was shown that type I fibers, which express SERCA2, have greater Ca^2+^ sensitivity of Ca^2+^ uptake compared with type II fibers, which express SERCA1 (Lamboley et al. 2014). Corresponding with this, when we compare WT diaphragm, a fast‐twitch muscle (our current study), with WT soleus, a slow‐twitch muscle (Fajardo et al. 2015), we find a much lower Ca^2+^ sensitivity of SERCA activity in diaphragm. While differences in SERCA isoform may contribute to the differences in *K*_*Ca*_ we have observed between diaphragm and soleus, they cannot explain the reduction in SERCA's apparent affinity for Ca^2+^ we observed in *Pln* ^OE^ diaphragm compared with WT since there were no differences in SERCA1 or SERCA2 between *Pln* ^OE^ and WT.

Upregulated SLN protein and/or mRNA is becoming widely known as a common feature in many myopathies (Nakagawa et al. 2005; Ottenheijm et al. 2008; Liu et al. 2011; Calvo et al. 2012) and findings in the *mdx* mouse and *mdx‐utrophin* double knockout model suggest that its expression may be directly proportional to disease severity (Schneider et al. 2013). Similarly, SLN may follow disease severity in the *Pln* ^OE^ mouse since the myopathy in the diaphragm, where only abnormalities in fiber CSA were evident, is far less severe than that found in the *Pln* ^OE^ soleus and gluteus minimus, where all histopathological signs of CNM with additional endomysial fibrosis and core‐like aspects were evident (Fajardo et al. 2015). However, the role that SLN plays in the *Pln* ^OE^ mouse model of CNM or any other animal model of myopathy remains unknown. Corresponding with the SERCA activity and Ca^2+^ uptake results, the maximal rates of relaxation and rates of contraction were not different between genotypes in the diaphragm, which is in direct contrast to what we and others have observed previously in the soleus of these mice (Song et al. 2004; Fajardo et al. 2015). However, we did find that the *Pln* ^OE^ diaphragm displayed a prolonged ½ RT compared with WT, which is consistent with the significant and trending reductions in SERCA's apparent affinity for Ca^2+^ and rates of Ca^2+^ uptake, respectively. Taken together, our results are suggestive of a relatively modest impairment in SERCA function in the *Pln* ^OE^ diaphragm, which, in part, may contribute to the lack of CNM histopathology in that muscle.

Analysis of hybrid fiber types further suggests that *Pln* overexpression in the diaphragm does not promote a type I fiber phenotype since the number of transitional type I/IIA fibers was not different between WT and *Pln* ^OE^ (Fig. S4A). Moreover, there were increases in type IIA/IIX and IIX/IIB hybrid fibers in the *Pln* ^OE^ diaphragm compared with WT; however, these effects only approached statistical significance (*P *=* *0.08 and 0.11, respectively; Fig. S4B and C). In any event, our data indicate that there was a general shift towards the faster fiber phenotype in the *Pln* ^OE^ diaphragm, which perhaps may explain the relatively small impairments in SERCA function and the lack in CNM phenotype. Analyses of central nuclei, fiber type distribution and CSA of 10–12‐month‐old mice produced similar findings to that of 4–6‐month‐old animals (Fig. S5), suggesting that the lack in CNM phenotype is not due to a delay in the disease progression of the diaphragm muscles.

To examine the underlying mechanisms behind these distinct effects on type I fiber proportions in the *Pln* ^OE^ diaphragm, soleus, and gluteus minimus muscles, we focused on the Ca^2+^‐dependent serine/threonine phosphatase, calcineurin. We suspect that in compensation for the type I fiber hypotrophy, the type II fibers of the postural soleus and gluteus minimus muscles exhibit greater load‐bearing activity thereby leading to myofiber hypertrophy and a fast‐to‐slow fiber type transition. A similar phenomenon is noted in functional overload studies whereby removal of the synergist muscles, soleus and gastrocnemius, causes the plantaris muscles to hypertrophy and transition towards a slow‐oxidative phenotype (Dunn et al. 1999; Michel et al. 2004). Importantly, calcineurin is activated during functional overload and is known to promote both the slow‐oxidative fiber phenotype (Timmerman et al. 1996; Dolmetsch et al. 1997; Chin et al. 1998) and myofiber hypertrophy (Dunn et al. 1999; Semsarian et al. 1999). Here, and similar to a previous study where calcineurin expression was found to be higher in the *Pln* ^OE^ soleus compared with WT (Song et al. 2004), our findings indicate greater calcineurin expression and NFAT nuclear content in the soleus and gluteus minimus muscles, but not in the *Pln* ^OE^ diaphragm (Fig. S6). Moreover, calsarcin‐2, an endogenous calcineurin inhibitor, is known to be highly expressed in diaphragm muscles (Frey et al. 2008). Thus, the inability to promote calcineurin signaling may help to explain both the reduction in the percent of type I fibers and the apparent increased susceptibility to muscle fatigue in the *Pln* ^OE^ diaphragm compared with WT. Although we did not assess force recovery after our fatigue protocol, successful recovery has been shown with a similar protocol (Coirault et al. 1999), which suggests that the protocol used in this study likely does not induce damage to the muscle fibers. Finally, it should also be noted that respiratory muscles do not typically undergo transformation with training and inactivity in the same way as limb muscle fibers, particularly with respect to MHC isoform (Polla et al. 2004). Thus, the reduced type I and increased type II fiber population may also represent a selective loss or underdevelopment of type I fibers due to PLN overexpression. Nevertheless, this innate response found within the diaphragm to limit the type I fibers may represent a novel therapeutic strategy combatting CNM and other congenital myopathies such as central cores since these are all type I fiber‐related myopathies (Sharma et al. 2009).

In summary, our results indicate that the *Pln* ^OE^ diaphragm, compared with soleus and gluteus minimus, is generally more resistant to the CNM phenotype, and that this lack in CNM pathology and weakness further supports the *Pln* ^OE^ mouse as a model of human autosomal dominant CNM where very little respiratory complications occur. Furthermore, we suggest that understanding the underlying mechanisms behind the diaphragm's apparent resistance to *Pln* ^OE^‐induced CNM and muscle weakness may lead to the generation of novel therapeutic strategies. Our findings here point to differences in SLN expression and type I fiber distribution as potential contributing factors, and future studies will determine their impact.

## Conflict of Interest

None declared.

## Supporting information


**Figure S1.** Lifespan of *Pln* ^OE^ (*n* = 23) and WT (*n* = 17) mice.

**Figure S2.** Monomeric (m) PLN expression in WT diaphragm is lower than WT soleus and gluteus minimus.

**Figure S3.** Ca^2+^ uptake assays in the soleus (A) and gluteus minimus (B) muscles from *Pln* ^OE^ and WT mice.

**Figure S4.** Percent distribution of hybrid I/IIA (A), IIA/IIX (B), and IIX/IIB (C) fibers in the diaphragm muscles from WT and *Pln* ^OE^ mice (*n* = 5 per genotype).

**Figure S5.** Analysis of central nuclei (A) and fiber type distribution (B) and cross‐sectional area (CSA; C) in 10–12‐month‐old WT (*n* = 4) and *Pln* ^OE^ (*n* = 5) mice.

**Figure S6.** Calcineurin (CnA) and nuclear factor of activated T‐cell (NFAT) nuclear content in soleus (A, D), gluteus minimus (B, E), and diaphragm (C, F) muscles from *Pln* ^OE^ and WT mice. (G) Nuclear cell fraction purity demonstrated through Western blots from tibialis anterior muscles using Histone H2B and CuZnSOD as nuclear and cytosolic markers, respectively.
