# Supplementary figures and images for: Diaphragm assessment in mice overexpressing phospholamban in slow‐twitch type I muscle fibers
Source: Brain Behav. 2016 Apr 22;6(6):e00470. doi: 10.1002/brb3.470 (PMC4842933; doi:10.1002/brb3.470)

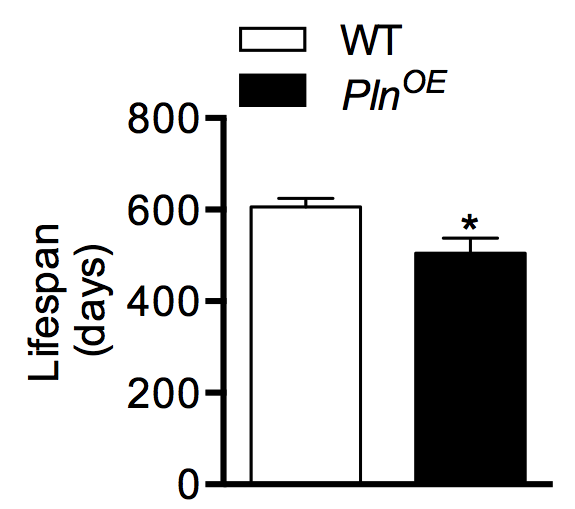

Supplement: Supplementary file 1 — Figure S1. Lifespan of Pln OE (n = 23) and WT (n = 17) mice. [file BRB3-6-e00470-s001.tiff]

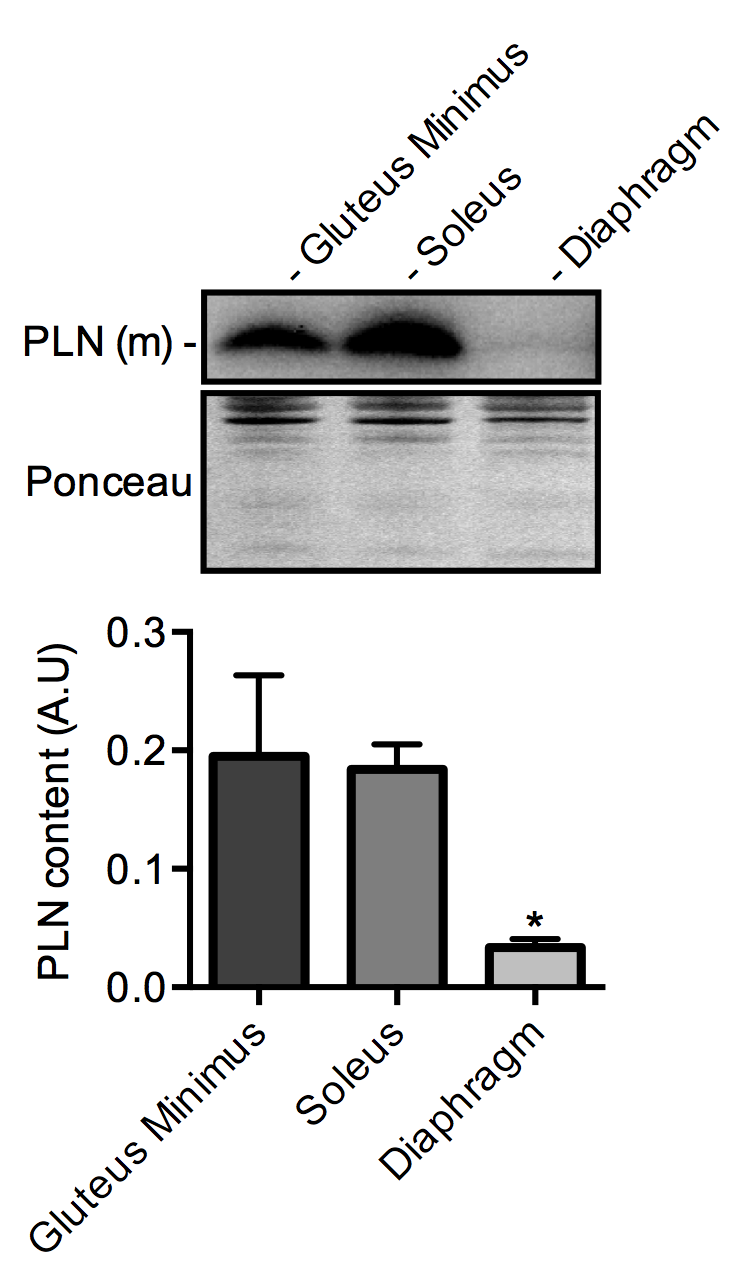

Supplement: Supplementary file 2 — Figure S2. Monomeric (m) PLN expression in WT diaphragm is lower than WT soleus and gluteus minimus. [file BRB3-6-e00470-s002.tiff]

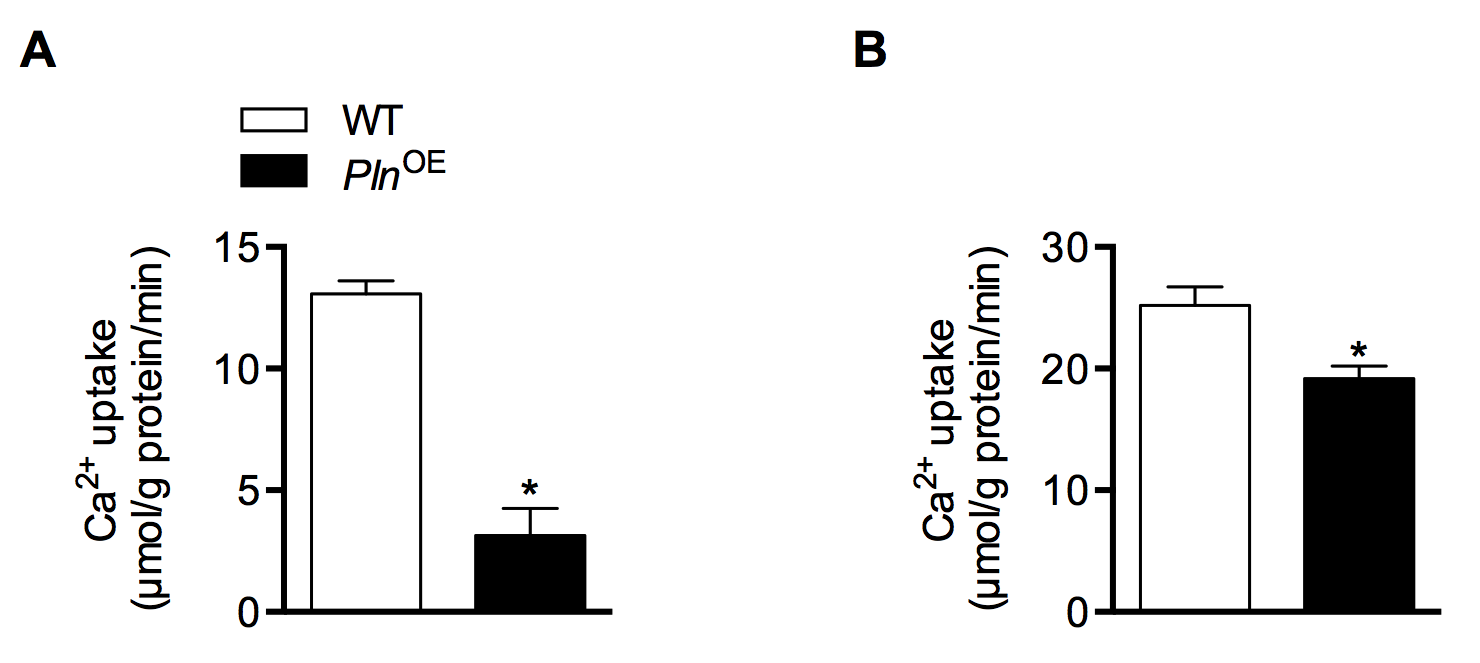

Supplement: Supplementary file 3 — Figure S3. Ca2+ uptake assays in the soleus (A) and gluteus minimus (B) muscles from Pln OE and WT mice. [file BRB3-6-e00470-s003.tiff]

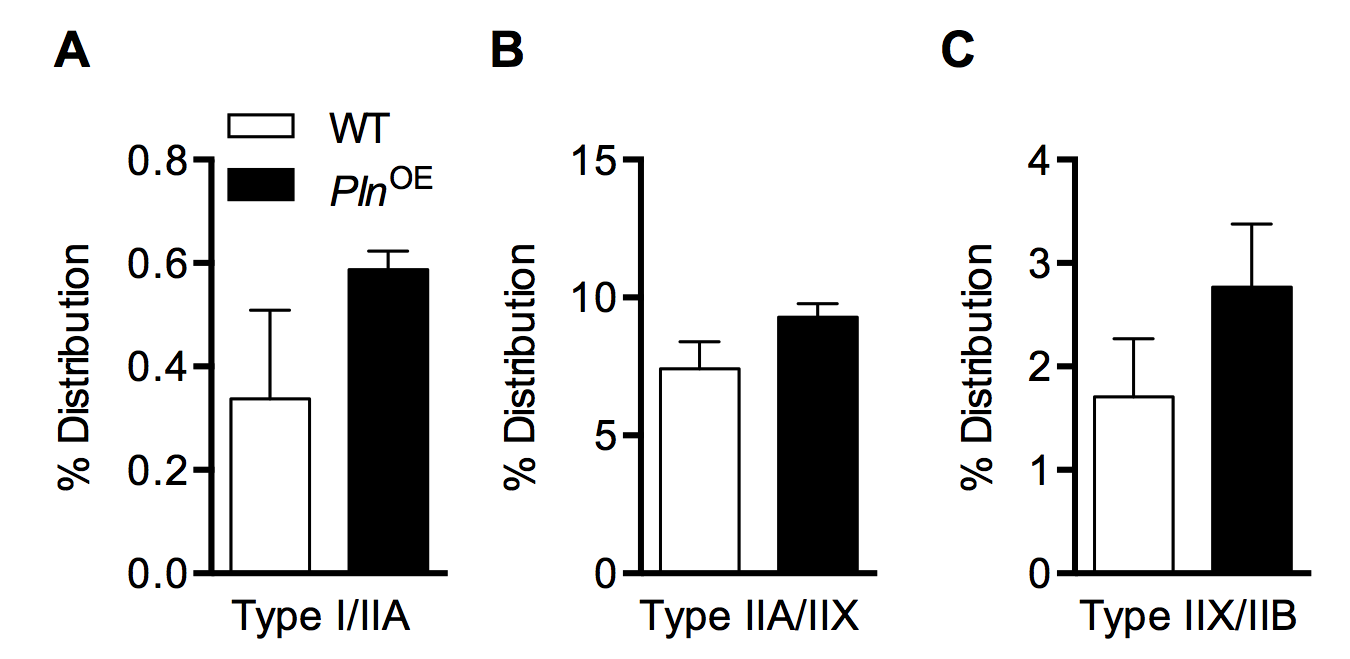

Supplement: Supplementary file 4 — Figure S4. Percent distribution of hybrid I/IIA (A), IIA/IIX (B), and IIX/IIB (C) fibers in the diaphragm muscles from WT and Pln OE mice (n = 5 per genotype). [file BRB3-6-e00470-s004.tiff]

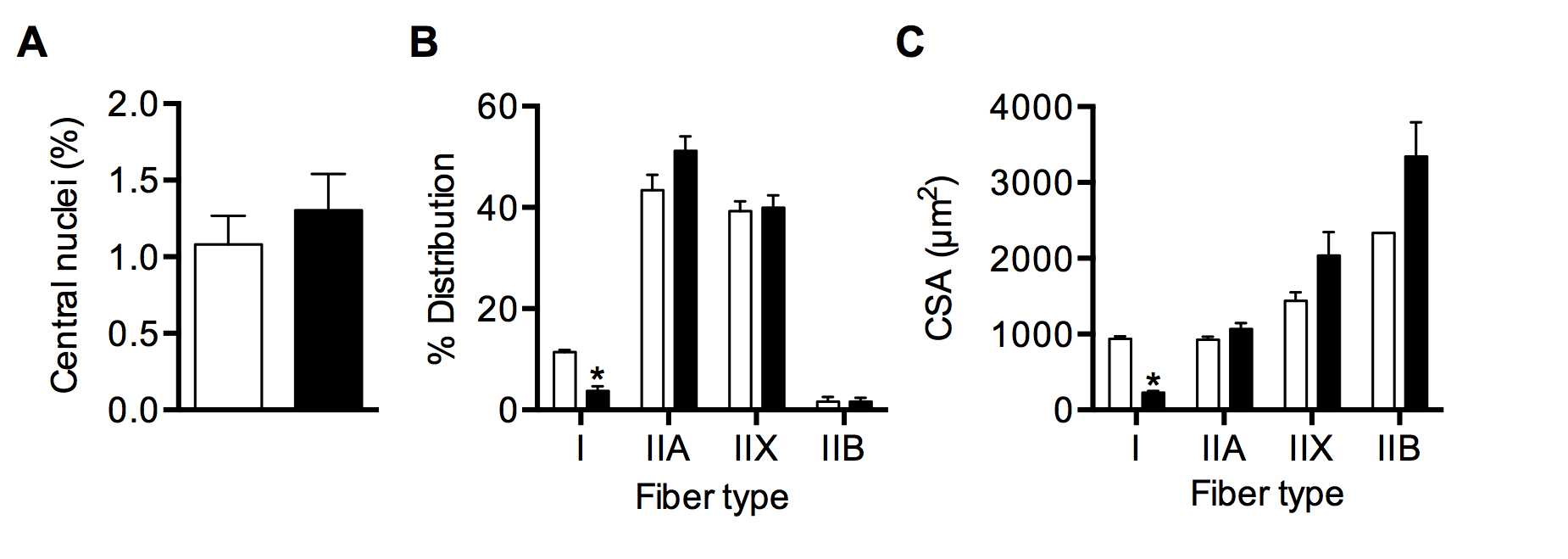

Supplement: Supplementary file 5 — Figure S5. Analysis of central nuclei (A) and fiber type distribution (B) and cross‐sectional area (CSA; C) in 10–12‐month‐old WT (n = 4) and Pln OE (n = 5) mice. [file BRB3-6-e00470-s005.tiff]

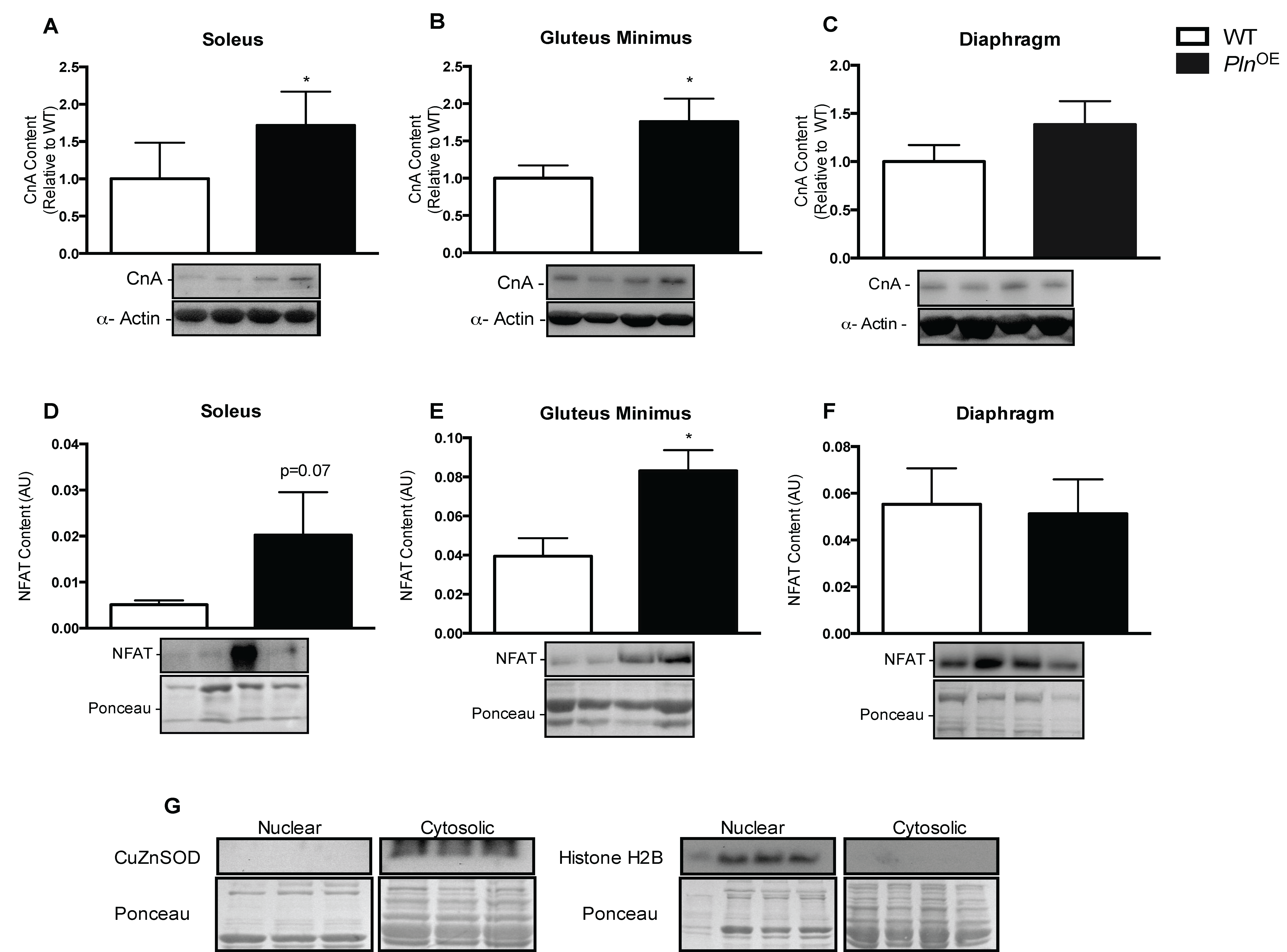

Supplement: Supplementary file 6 — Figure S6. Calcineurin (CnA) and nuclear factor of activated T‐cell (NFAT) nuclear content in soleus (A, D), gluteus minimus (B, E), and diaphragm (C, F) muscles from Pln OE and WT mice. (G) Nuclear cell fraction purity demonstrated through Western blots from tibialis anterior muscles using Histone H2B and CuZnSOD as nuclear and cytosolic markers, respectively. [file BRB3-6-e00470-s006.tiff]
